# Relationship Between the Intersesamoid Ligament and Sesamoid Bones in Cadaveric Feet with Hallux Valgus

**DOI:** 10.7759/cureus.1819

**Published:** 2017-11-03

**Authors:** Regina C Fiacco, Garen M Ream, Charlotte Wilson, R. Shane Tubbs, Marios Loukas, Piotr B Kozlowski, Anthony C DiLandro, Kevin T Jules, Anthony V D'Antoni

**Affiliations:** 1 New York College of Podiatric Medicine; 2 Seattle Science Foundation; 3 Neurosurgery, Seattle Science Foundation; 4 Department of Anatomical Sciences, St. George's University School of Medicine, Grenada, West Indies; 5 Neurology, Neuromedlab; 6 Division of Pre Clinical Sciences, New York College of Podiatric Medicine; 7 Department of Surgery, New York College of Podiatric Medicine

**Keywords:** anatomy, intersesamoid ligament, hallux abducto valgus, bunion, surgery, morphology

## Abstract

There is heterogeneity in the literature regarding the anatomy, exact location, and definition of the intersesamoid ligament (IL). Anatomic knowledge of the IL and its variations are important for surgeons who undertake corrective surgery for hallux abducto valgus (HAV). The IL was dissected in 16 feet from 32 formalin-fixed cadavers (12 females, four males; mean age at death, 76.6 years) to examine its morphology. The length, width, and thickness of its constituent bands were recorded with a digital caliper. Descriptive and correlational statistics were used to investigate the relationships between band size, age at death, and sex. A literature review was conducted to compare our data to those of previous studies. Results suggest that the size of the sesamoids may change as a result of HAV deforming forces, which may cause lengthening of the IL. The IL stabilizes the sesamoid apparatus of the first^ ^metatarsophalangeal joint (MPJ) and should be evaluated in HAV correction. Anatomic knowledge of the complex morphology and relations between the IL and sesamoids is critically important for surgeons correcting HAV deformities.

## Introduction

The intersesamoid ligament (IL) of the first metatarsophalangeal joint (MPJ) is an essential structure that bridges both tibial and fibular sesamoids of the hallucal sesamoid complex [1-4]. Hallux abducto valgus (HAV) encompasses sesamoidal deviation, malalignment, and metatarsal rotation, all of which act at the capsuloligamentous structure of the 1st MPJ [2-3, 5-7]. The IL is also a major stabilizer of the base of the proximal phalanx and counters shear forces from about 60% of a person’s body weight [5-6]. These functions suggest that the IL plays a major role in preventing HAV.

HAV is a complex deformity that includes lateral deviation of the hallux, medial deviation of the first metatarsal head, and lateral deviation of both sesamoids [6]. HAV prevalence is estimated at 23% of adults aged 18 to 65 years and in 35% of adults over age 65. HAV increases with age and is more prevalent in females [8]. Detailed anatomic awareness of the exacerbated effect of IL on sesamoidal deviation is important to consider in surgical correction of HAV.

There is a current lack in normative cadaveric data that solely pinpoints the dimensions of the IL. Instead, the literature has grouped the majority of the ligamentous structures of the first MPJ as a whole. These data have identified the anatomy of the MPJ, but have yet to describe findings using morphometric and statistical analyses [1-3, 6-7]. There is also an absence of studies correlating the difference in IL length and the prevalence of HAV.

The IL has been anatomically described as a ligamentous structure that connects both medial and lateral sesamoid bones of the first metatarsophalangeal joint [9]. Additionally, the flexor hallucis longus tendon is juxtaposed to the plantar surface of the intersesamoid ligament [3]. Confusion is apparent in the literature when differentiating between the plantar plate and IL. Some papers refer to the IL as the central portion of the plantar plate [4]. However, the more commonly accepted description is that the IL is more dorsal to the plantar plate [1-3]. It is necessary to conduct studies to distinguish the IL from the other structures in the hallucal sesamoid complex.

HAV causes medial translation of the metatarsal head, which leads to the proposal that although the IL stabilizes the sesamoids, a constant overbearing force may lengthen the IL [6]. The purpose of this cadaveric study was to examine and statistically analyze the morphology of the IL. This study was driven by the paucity of anatomic data on the pathologic changes that affect the IL and sesamoids in patients with HAV. This understanding will foster future research to specifically focus on how clinical implications of HAV can cause a significant length differentiation of the IL. Ultimately, this relationship can create a new tool for correcting HAV in patients.

## Materials and methods

Thirty-two formalin-fixed feet from 16 cadavers (12 females, four males) were dissected (16 right and left) from both dorsal and plantar aspects to expose the IL. All of the specimens were extracted using blunt and sharp dissecting tools in an effective manner to expose the intersesamoidal ligament. Proper technique involves many steps including dissection from the plantar aspect through the skin, fat, and fascia at the level of the first MPJ. The tendon of flexor hallucis was maintained and moved to the side to expose the plantar plate. Careful dissection through the plantar plate allowed the ligament to be visualized from outside the first MPJ. Following the measurement of all external structures, we then opened the joint capsule to visualize and measure the ligament from within the first MPJ. Length, width, and thickness of IL and sesamoids were measured with a digital caliper (Hawk, Inc., Cleveland, Ohio). Total length and width of each foot were measured. Measurements were compared in non-hallux valgus (HAV) feet and HAV feet. HAV feet were verified by observation. The data was analyzed using Statistical Package for the Social Sciences (SPSS Version 20.0, IBM, Armonk, NY). Descriptive and correlational statistics were used. A P-value of less than 0.05 was considered significant. Histology was performed on certain specimens to verify ligamentous structures. Digital radiographs of the cadaveric HAV feet were taken using a model 715 A-BD x-ray unit (X-CEL X-ray, Inc., Crystal Lake, IL).

## Results

**Figure 1:**
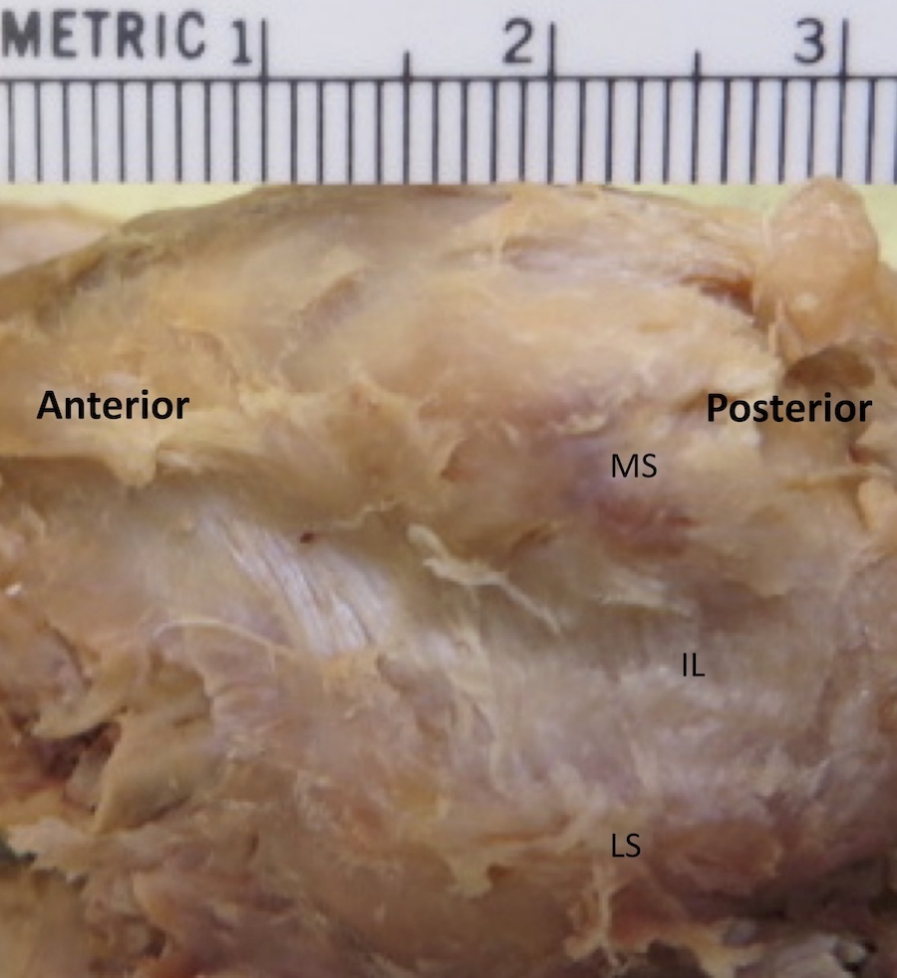
Image of a cadaveric right foot highlighting fibers of the intersesamoid ligament from a plantar view IL: Intersesamoid ligament MS: Medial Sesamoid LS: Lateral Sesamoid

From the plantar aspect (outside capsule), the IL mean (SD) length was 8.66 (1.21) mm, and the mean width (SD) was 6.27 (1.48) mm (Figure 1). From the dorsal aspect (in capsule), the IL mean (SD) length was 6.47 (0.85) mm, and the mean (SD) width was 2.24 (0.61) mm. Mean (SD) thickness from dorsal to plantar (DP) was 3.03 (0.65) mm. Five of 32 (15.6%) feet had HAV. Independent-samples t-tests between non-HAV and HAV feet revealed the following significant differences for left feet: medial sesamoid length (plantar) (P=.002) and lateral sesamoid length (dorsal) (P=. 020). For right feet: medial sesamoid length (dorsal) (P=.003), and plantar (P=.014), with lateral sesamoid length (dorsal) (P=.004). For both feet (total): medial sesamoid length (dorsal) (P=.008) and plantar (P=.035). There is a moderately strong direct relationship between IL length and lateral sesamoid width when measured from a dorsal aspect. There is a strong direct relationship between IL length (measured from a plantar aspect) and medial sesamoid width (measured from both plantar and dorsal aspects). The data suggests that the total length of the medial and lateral sesamoids increase in HAV feet.

## Discussion

The severity of HAV has been described as either mild, moderate, or severe, based on weight-bearing radiologic assessments [10]. Determination of HAV severity is reliant on two angles: the hallux valgus angle (HVA) and the intermetatarsal angle (IMA). For the HVA, normal is considered <15°, mild is <20°, moderate is 20°- 40°, and severe is >40°. For the IMA, normal is considered <9°, mild is ≤11°, moderate is <16°, and severe is ≥16° [10].

The IL is the main connection between the medial and lateral sesamoid bones. It must be cut to remove either the medial or lateral sesamoid following trauma and is often severed during severe HAV surgery to correct a bunion deformity of the first MPJ. We found that the mean length of the IL was 8.66 mm, and the mean width was 6.27 mm. From the dorsal aspect (in capsule), the mean length of the IL was 6.47 mm and the mean width was 2.24 mm. The mean thickness from dorsal to plantar was 3.03 mm. Due to the connection between the sesamoids via the IL, in HAV surgery, reapproximation of the sesamoid complex underneath the head of the first metatarsal is important for biomechanical stability. If conservative efforts are unsuccessful, surgery is the recommended next option. The most common procedure is the chevron osteotomy due to its effectiveness and consistent results [11].

## Conclusions

Currently, surgical correction usually involves transection of the IL to allow removal of the lateral sesamoid in severe deformities. The length and width of the IL is the key factor in determining the distance between the sesamoid bones, and our results suggest that the size of the sesamoids may change as a result of HAV, causing lengthening of the IL. Further studies are needed to better understand the role of the IL and sesamoids in HAV deformity utilizing a larger sample size. Anatomic knowledge of the complex morphology and relations between the IL and sesamoids is critical for surgeons correcting HAV deformities due to the deviation of the sesamoid complex, and the resultant pathologic changes.
